# Upper Gastrointestinal Tract Transit Times of Tablet and Drinkable Solution Formulations of Alendronate: A Bioequivalence and a Quantitative, Randomized Study using Video Deglutition

**DOI:** 10.1007/s00223-012-9639-9

**Published:** 2012-08-26

**Authors:** Claudia Gómez Acotto, Carlos Antonelli, Damien Flynn, Dennis McDaid, Emilio J. A. Roldán

**Affiliations:** 1 Department of Phosphocalcium Metabolism, Maimónides University, Buenos Aires, Argentina; 2 Department of Diagnostic Imaging, Maimónides University, Buenos Aires, Argentina; 3 Xeolas Pharmaceuticals Ltd, Dublin, Ireland; 4 Departamento Fosfocálcico, Universidad Maimónides, Hidalgo 577, Ciudad Autónoma (C1405BCG) de, Buenos Aires, Argentina

**Keywords:** Alendronate, Bioequivalence, Digestive transit, Liquid formulation

## Abstract

The bioequivalence and upper digestive tract transit time of a drinkable solution of 70 mg/100 mL alendronate was compared to reference tablets. A randomized, single- dose, two-way crossover study of the rate of urinary recovery of alendronate during 36 h (AE_(0–36 h)_) by HPLC, in 104 healthy young male volunteers, showed that AE_(0–36 h)_ and the maximum excretion rate (*R*_max_) were within the accepted range of bioequivalence 81.8–105.7 and 81.7–106.2, respectively. To characterize the oesophageal passage time of the two alendronate formulations, we performed a randomized, controlled study, in 24 healthy men and women (mean 52 years old), who took the formulations standing or lying down, by an X-ray video deglutition system. When taken in the standing position, both formulations had equal mean transit times from mouth to stomach and tablet disintegration but data dispersion was significantly smaller with the liquid form. When taken in lying position, drinkable alendronate had shorter and less variable median transit times compared to the tablets. These results show that the drinkable alendronate formulation is bioequivalent to the tablets and may be advantageous in patients in whom the transit or disintegration of the tablets is impaired.

## Introduction

Orally administered bisphosphonates have very critical absorption profiles that depend on the postdosing fasting period, which is usually recommended to be at least 30 min. In practice, however, not all patients adhere to this recommendation, thereby reducing the absorbed fraction of the drug and jeopardizing the outcome of long-term treatments [1–5]. The critical dependence of bisphosphonate action on the postdosing fasting period is related to the chemical and physical properties of these molecules and their mechanism of absorption.

Bisphosphonates are poorly soluble drugs that cannot cross the intestinal barrier through cell membranes; many molecules most probably remain trapped by calcium-containing proteins in the intercellular space [6]. Absorption begins when the irritant effect of the molecules causes local edema, transiently broadening the intercellular spaces and thus enabling a small portion of the soluble bisphosphonate molecules to reach the bloodstream [6]. This intricate absorption mechanism is the reason for the low bioavailability, and local irritation accounts for the typical digestive discomfort frequently reported by patients taking oral bisphosphonates [7–11].

The convenience of triggering this irritant mechanism intermittently—once weekly or once monthly rather than daily—is the rationale for the dosing regimens currently in use [12]. At the same time, however, the risk of diminished therapeutic response due to impaired absorption increases proportionally with the intermittent schedules of administration. Consequently, ensuring that soluble bisphosphonate molecules are available at the site of absorption within 30 min after dosing is essential for therapeutic success in clinical practice.

Alendronate is currently the most commonly used agent for the treatment of osteoporosis. Experimental pharmacokinetic studies show that absorption can occur in the stomach and also in the first portion of the small intestine [13–15]. Quick delivery of the drug to the intestine is therefore expected to be accompanied by fewer upper digestive tract symptoms. The oral solution affords soluble alendronate in a drinkable formulation of pharmaceutical quality, at a concentration below 1 % -which is not irritant- and circumvents certain problems described with the solid formulations [16], for instance: adherence of the tablet to the digestive mucosa; the challenge to overcome potential motility obstacles such as hernia, spasm, the body position of the patient during transit; a slow, variable rate of disintegration which causes precipitation or reflux of irritant particles, acidity [7–10]. The alendronate that is retained in the stomach during the postdosing fasting period stimulates absorption through the gastric walls, provided it is not exposed to interaction with food or with the high mineral contents of the water [17–21].

The current intermittent schedule of alendronate administration makes the use of drinkable forms more comfortable for patients on long-term treatment for osteoporosis [12].

In view of the above, we studied the transit in the upper digestive tract of alendronate given either as tablets or as drinkable solution in a group of healthy adults, with the aim to obtain quantitative data on the differences between the administrations of the two formulations that might impact its use in clinical practice. Before this study, we tested the bioequivalence of the two formulations according to current regulatory requirements.

## Subjects and Methods

### Bioequivalence Study

A randomized, single-dose, fasting, two-way crossover study tested the pharmacokinetic bioequivalence of fast disintegrating alendronate tablets and a drinkable alendronate solution in subjects recruited at the MTZ Clinical Research site in Warsaw. The study was conducted according to the World Medical Association Declaration of Helsinki and current good clinical practice guidelines and state regulations of Poland. The study protocol was approved by the Bioethics Committee of the Warsaw Regional Chamber of Physicians and Dentists and by the European Regulatory Authority.

The reference product was the 70 mg Fosamax tablet manufactured by Merck Sharp & Dohme Limited, UK, and the test product was a 70 mg/100 mL drinkable alendronate solution with thickening agents and orange color and flavor (Xeolas Pharmaceuticals Ltd., under the license of Gador SA). The active ingredient of this formulation has already been described [22].

Prospective participants were informed about the study and then asked to sign an informed consent form prepared according to MTZ’s standard operating procedures. Those who consented were examined and eventually 108 healthy men were accepted into the study, of which 104 successfully completed all stages of the protocol. There were four withdrawals: one subject withdrew voluntarily, two subjects experienced adverse events (one had increased blood pressure during period 1 and another one reported an adverse event not related to the study products during the washout period), and another subject was tested positive for unauthorized drugs. Main inclusion criteria were: male gender, age 18–50 years, and body mass index between 19.0 and 26.0 kg/m^2^. In addition to other routine criteria, subjects were required to be able to refrain from smoking 3 days before the beginning of the study until its completion. Exclusion criteria: current or chronic health problems and/or drug therapy; a history of allergy, hypocalcemia, digestive, hepatic or renal disorders; a history of excessive alcohol consumption (>30 g/day during the previous year); positive test results for anti-HIV, HBsAg or anti-HCV. In addition subjects with abnormal blood pressure, pulse or laboratory test results were excluded from the study.

Participants were randomized according to a numerical table and were given either a tablet to be swallowed with 240 mL of water or a drinkable solution to be swallowed with 140 mL of water, to ensure equivalent hydration. No additional water intake was allowed from 1 h before to 1 h after dosing, but subjects were afterward administered 2,000 mL of fluids according to a standardized distribution and a 2,500–3,000 kcal/day diet divided in three meals, given at 4, 9 and 12 h after dosing.

After a 14 day washout period, study participants were asked to return to the research center to be given the other alendronate formulation. On each of the two dosing days, urine samples were collected at the following time points: 1–1.5 h before dosing, and then after dosing during the following time intervals: 0 to 0.25; 0.25 to 1 h; 1 to 2 h; 2 to 3 h; 3 to 4 h; 4 to 6 h; 6 to 8 h; 8 to 12 h; 12 to 24 h and 24 to 36 h. Each urine sample was collected into a container from which two 6 mL aliquots were drawn and placed into plastic tubes. Samples were frozen (−20 °C) and were sent to BioClin Research Laboratories Ltd. (Ireland), where urinary alendronate concentrations were determined under blind conditions using a HPLC–fluorescence method (BioClin test method number PR120) which involves the isolation of alendronate from human urine by solid-phase extraction. Alendronate was coprecipitated with calcium phosphate and then its primary amino group was derivatized with 2,3-naphtalene dicarboxyaldehyde (NDA) and N-acetyl-d-penicillamine (NAP) to form the fluorescent derivative. A Shimadzu LC-10AS (Masons, Dublin) system, a Waters 717 Plus autosampler (Waters, Dublin) and a Waters 474 fluorescence detector were used for these measurements. Intra-assay precision (%CV) was in the 3.95–8.05 % range and mean percentage accuracy in the 105.5–112.5 % range. The precision at the lower limit of quantitation (10 ng/mL) was 5.43 % (defined by %CV), with a 100.1–115.4 % accuracy. Interassay precision ranged from 4.37 to 14.1 % and mean accuracy ranged from 105.9 to 112.8 %. The method was validated in the concentration range 10–1,000 ng/mL.

The primary evaluation parameter was the total amount of drug excreted in the urine from the time of dosing until 36 h after dosing (total AE_(0–36 h)_) and the maximum urinary excretion rate (*R*_max_). Secondary parameters were the amount of drug excreted (AE) and the rate of urinary excretion (*R*e) in each collection interval, and the time of maximum urinary excretion rate (*T*_max_). Descriptive statistical analysis was performed and Lund’s method was used for outlier detection.

The log-transformed total AE_(0–36 h)_ and *R*_max_ were statistically compared by ANOVA analysis of variance considering the effect of the treatments, sequence and period of study. The 90 % confidence interval was tested by LSMEANS for AE_(0–36 h)_ for the 80–125 % range, and by Hodges-Lehmann nonparametric methods for *R*_max_ for the 75–133 % range. WinNonlin version 4.0.1. was used for the kinetic parameters and SAS version 9.1 for the statistical calculations.

In order to assess safety, adverse events observed during the study were reported (using CTCAE v. 3.0 software) and clinical examinations, including the following measurements and procedures, were performed before and after each dosing period: pulse, blood pressure, body temperature, ECG, X-rays, and clinical laboratory determinations such as urine analysis and hematology, biochemistry, serology and controlled substance (alcohol, opioids, barbiturates, benzodiazepines, amphetamines, cocaine, cannabis and nicotine) tests.

## Video Deglutition Study in Healthy Adults

This was a prospective, randomized, controlled trial comparing the upper digestive tract transit of two alendronate formulations, conducted according to good clinical practice guidelines. The study protocol was approved by the Institutional Ethics Committee of the Maimónides University and by the National Administration of Drugs, Food and Medical Technology (ANMAT, appl. 4906). After a period of training in procedures such as subject positioning and X-ray follow-up of the formulations, the study was opened. All subjects provided signed informed consent. Twenty-four healthy adult volunteers, mean age 51.6 years (range 39–68 years), were recruited and randomized according to an age-cohort table in order to obtain a balanced distribution of ages within the range. Patients were excluded if: there was clinical evidence of any chronic disease; they were hypersensitive to bisphosphonates; they consumed aspirin, other nonsteroidal anti-inflammatory drugs and/or alcoholic beverages on a regular basis; or they had experienced a bone fracture or taken part in any other study during the 60 days before this study. Both tested formulations were the same as in the previous study. In order to facilitate the X-ray follow-up, the tablets were drilled with a fine drill and partly refilled with contrast substance, and 5 mL of the 100 mL drinkable solution were replaced by contrast media. The in vitro disintegration test of the original and the modified tablets did not show any significant differences in the dissolution time (data not shown).

**Fig. 1:**
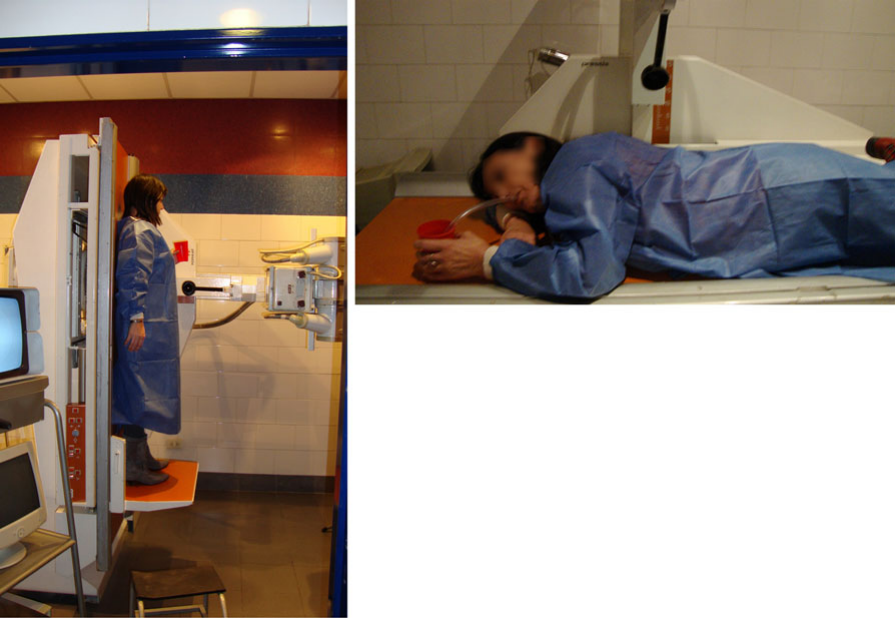
Body position of the participants during the upper digestive tract transit study. *Left* standing position. *Right* bed rest (prone) position

Participants were given either the tablet or the drinkable solution standing or lying in prone position (Fig. 1). Each subject participated randomly in 3 out of 4 possible experiments. The formulations were administered under fasting conditions, which were maintained until the subjects had finished the three series of experiments. Subjects held the required body position for at least 20 min after dosing.

All participants were trained to comply with the study procedures. From a few minutes before dosing until the experiment was completed, subjects were positioned in front of a remotely controlled X-ray system of 750 mA, 150 kV, with a high-resolution magnifier and digitizer (Pinnacle DC 1000 MPEG 2, stored in a HPPavilion Dv1025IA personal computer), operated by a skilled radiologist. All procedures were recorded and filed for analysis.

In the video analysis, only subjects whose images were clear for the entire sequence were considered. For statistical purposes, a mouth to stomach time (MST) of 480 s was considered for tablet disintegration before reaching the stomach and a disintegration time (DT) of 20 min for nondisintegration. After the tablet with water and the drinkable solution had been completely swallowed, MST was compared for both formulations. The arrival of the tail of the swallowed volume was considered, and the drinkable solution was followed until the head of the volume was clearly detected in the first portion of the duodenum (mouth to duodenum time, MDT). The tablets were followed until they lost their shape—they typically formed a disintegration cloud—and disappeared (i.e., DT), whether this occurred in the esophagus, the stomach or the small intestine.

After the images had been digitalized, a PC time counter was used to estimate MST for both formulations, which were compared for the standing or upright and horizontal positions by analysis of the mean and the variance values by *t* test for independent groups. Variance (S) is a measure of how robust the mean value is as a function of the distribution of the individual values considered. Likewise, MDTs for the drinkable solution and DTs for the tablet formulation were compared for the two body positions of the subjects by *t* test for dependent samples. Median and ranked paired analysis by Wilcoxon test were also performed. Nonparametric Spearman rank order correlation test, with pairwise deletion of cases was used to associate MST, MDT and DT with the age of the participants, and within treatments.

Once the experiments were over, the subjects remained in the Unit under clinical supervision for 2 h and were then discharged and instructed to come back or call should they experience any adverse events or discomfort during the next week. During this period, patients were called back for a new visit if any anatomic or functional abnormalities were detected in the video deglutition images.

## Results

### Bioequivalence Study

The study protocol was successfully completed by 104 subjects, aged 26.7 ± 7.9 years, with a body mass index of 23.2 ± 2.1. Table 1 shows the main results for the pharmacokinetic variables studied. When data for both alendronate formulations were compared, AE_(0–36 h)_ and *R*_max_ were found to be within the acceptable range for bioequivalence, respectively 81.8–105.7 and 81.7–106.2. Intrasubject variation for AE_(0–36 h)_ was 60.3–61.8 % for *R*_max_; the statistical power of the sample was 0.89 and 0.88, respectively, which is sufficient for establishing bioequivalence.

**Table 1:** Pharmacokinetic parameters from a bioequivalence study comparing single 70 mg dose taken either via a tablet or a drinkable solution (0.7 %) in 104 healthy young men after an overnight fast and a 4 h postdosing fasting period

Formulation	Tablet	Drinkable formulation	BE (IC_90_)
Alendronate urinary excretion after a single 70 mg administration			
AE_(0–36 h),_ μg, mean (SD)	167.3 (147.3)	140.7 (109.9)	
AE_(0–36 h)_, μg, median (min–max)	124.2 (8.8–818.8)	121.9 (32.1–919.9)	
AE_(0–36 h)_, %CV	88.1	78.1	81.8–105.7
*R*_max_, μg/mL, mean (SD)	54.4 (50.3)	47.3 (37.4)	
*R*_max_, μg/mL, median (min–max)	41.9 (4.6–334.7)	37.1 (5.03–266.34)	
*R*_max_, %CV	92.5	79.1	81.7–106.2
*T*_max_, h, mean (SD)	1.58 (0.67)	1.68 (0.72)	
*T*_max_, h, median (min–max)	1.5 (0.13–3.5)	1.5 (0.63–5.0)	

Bioequivalence between both formulations is within the acceptable range

**Fig. 2:**
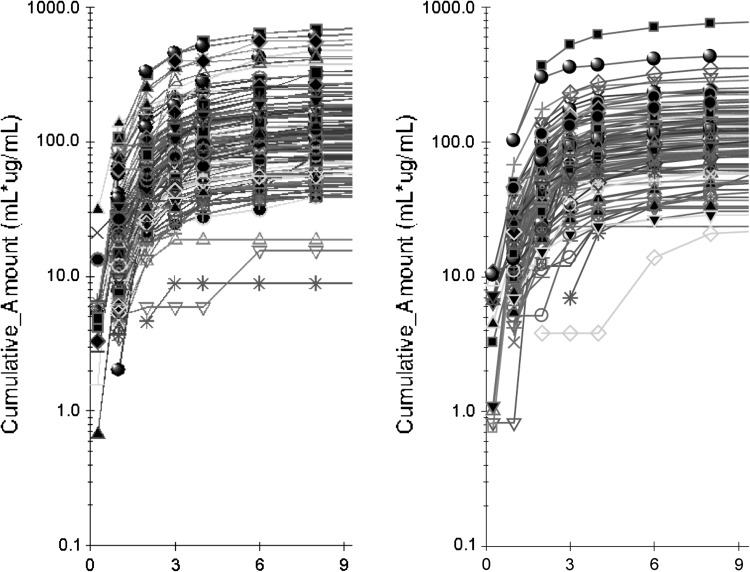
Individual curves of the cumulative amount of alendronate excretion in urine in 104 healthy young men after receiving, in fasting conditions (>8 h before and 4 h after administration), a single 70 mg dose in a tablet +240 mL of plain water (*left*), or a single 70 mg/100 mL drinkable solution +140 mL of plain water (*right*); truncated at 9 h after dosing. From hours 9–36, the cumulative amount is minor; the shape of the curves does not change

Median AE_(0–36 h)_ was very similar for both products; 121.9 μg for the drinkable solution and 124.4 μg for the tablets; mean values were higher, especially for the tablets, which also showed a greater SD. %CV was 78.1 % for the drinkable solution and 88.1 % for the tablets (ie, 12.8 % higher for the tablets). The distribution of the number of cases according to AE_(0–36 h)_ showed that in the 112.5–212.5 μg range, which represents the magnitude of urinary alendronate excretion found with the highest frequency (63 of 104 for the drinkable solution and 52 of 104 for the tablets), there were 21.2 % more subjects treated with the drinkable solution than with the tablets (Fig. 2). On the other hand, the distribution of cases treated with tablets tended to be higher in subjects with the lowest (<112.5 μg) or highest (>212.5 μg) levels of urinary recovery. Above 412.5 μg, 9 patients were treated with tablets and 3 with the drinkable solution. Median *R*_max_ values were 37.1 μg/mL for the drinkable solution and 41.1 μg/mL for the tablets. Variations in *R*_max_ show a similar pattern, with %CV being 16.9 % higher for the tablets; this parameter, however, is less clinically relevant for this type of product [23].

Mean urinary alendronate concentration did not differ significantly between the two formulations at any of the collection periods. For example, during the first hour urinary alendronate concentration was 109.0 ± 302.5 μg/L after the tablets and 177.3 ± 205.4 μg/L after the solution (not significantly different). However, the variance was significant lower with the solution (*F* test, *p* < 0.05). Overall, 62 nonserious adverse events were reported as mild or moderate, with the exception of one case of severe headache, which resolved without sequelae. Forty-one of these events occurred during period 1 (in 30 of 106 subjects) and 21 during period 2 (in 17 of 104 subjects). The distribution of adverse events was similar for the tablet and for the drinkable solution; 31 events were reported for each formulation. Of these none was considered by the investigators to be definitely related to the products. Five were classified by the investigators as probably product related; 2 (headache and nausea) with the tablet and 3 (2 cases of headache and 1 with diarrhea) with the solution. Other 23 events were classified possibly product related, 14 with the tablet: headache (*n* = 6), hypophosphatemia (*n* = 3), hyperbilirubinemia (*n* = 2) and hypocalcemia, myalgia and pain of knee (*n* = 1 each), and 9 with the solution hypophosphatemia (*n* = 4), headache (*n* = 2), myalgia (*n* = 2) and vomiting (*n* = 1). The type and frequency of these events are consistent with those described in the summary product characterization of Fosamax.

## Video Deglutition Study in Healthy Adults

Videos were examined after completion of all procedures. Data from one subject were discarded because the contrast material that remained after the first experiment precluded an adequate follow-up of all experimental sequences. In two subjects—a 66-year-old woman and a 68-year-old man—previously unknown hiatus herniae were detected, which did not affect transit times for either the tablet or the drinkable solution. A 68-year-old woman from the bed rest position group showed difficulties in the passage of the tablet through the esophagus and quick disintegration once the tablet had reached the stomach. In a 60-year-old man who took the tablet in the standing position, the tablet stuck to the esophagus and disintegrated there. Early tablet disintegration in the esophagus upon swallowing the formulation in the bed rest position was also observed in a 52-year-old man. Neither of these two subjects met the MST criteria. The video of a 52-year-old man revealed that the tablet had remained in the esophagus for 8 min, then passed intact through the stomach and the pylorus and did not disintegrate at any time during the 20 min video follow-up. A 51-year-old woman whose images showed a “waterfall” stomach had a very short MST, both with the tablet and the drinkable solution. The same was observed in a 40-year-old woman with a similar gastric image. In the video of a 46-year-old woman, a diverticulum was detected in the lower portion of the esophagus, and because the subject had initially been administered the drinkable solution formulation, the subsequent images were not clear enough for calculating the tablet disintegration time.

Table 2 summarizes the mean, median, variance and range values for the estimated MST, MDT and DT. Mean MST was very short for the two formulations; although the drinkable solution proved to be the one with the shortest transit time, the differences between the two formulations were not statistically significant when dosing under either of the body positions studied (Table 3). However, the variance and range were larger for the tablets than for the liquid formulations. When the tablet transit time is assessed, medians are smaller than means (Table 2), which shows that the distribution curve does not behave normally as a result of the high dispersion of the data (S) with tablets. Therefore, the ranked analysis (Wilcoxon test Table 3) of the MSTs shows that the MST for the drinkable solution administered in the standing position is significantly shorter than for the tablet (standing and prone position) or the drinkable solution taken in the prone position, even though they all have the same median value (5 s), which suggests that delays are more likely to occur when dosing in the prone position. Likewise, the drinkable solution is also significantly faster than the tablet when dosed in the prone position. Mean and median MST measured for the standing position are faster for the drinkable solution but the sample size does not provide enough power for statistical significance between the parameters. Conversely, the analysis of the variances did have statistical significance, showing that variability in transit was much smaller after taking the drinkable solution, for both body positions studied. This is determined by the early disintegration of the tablet in the esophagus or by a delay in its passage to the stomach in some cases, which enhanced variance to *S* = 12,567.5 s^2^ in the group of subjects who took the tablet while standing, and to *S* = 29,715.1 s^2^ in the bed rest group. For the tablet, the differences in body position upon dosing were not significant in this small sample, and the variance was double (*p* < 0.09), which suggests that this trend could be confirmed with a larger sample. Body position upon dosing clearly did not affect the transit of the drinkable solution to the stomach (Tables 2, 3). The variance for the drinkable solution taken in the bed rest position is smaller than for the tablet, both for the standing and the bed rest positions. The low *p* values may suggest a trend in differences.

**Table 2:** Transit time from mouth to stomach (MST), mouth to duodenum (MDT) and disintegration time (DT) for a tablet plus 250 mL of plain water and for 100 mL of a drinkable solution containing 70 mg of alendronate, assessed by X-ray video-deglutition analysis in groups of healthy adults

Administration	MST, s				MDT, min				DT, min			
	Mean (*n*)	Median	Variance, s^2^	Range	Mean (*n*)	Median	Variance, s^2^	Range	Mean (*n*)	Median	Variance, s^2^	Range
Tablet, standing position	30.9 (18)	3.8	>999.9	2.0–480.0	NA				3.8 (17)	3.5	13.6	0.8–17.0
Tablet, bed rest position	73.1 (14)	5.0	>999.9	3.0–480.0	NA				3.4 (15)	2.3	22.7	0.5–>20
Drinkable solution, standing position	6.8 (18)	5.0	72.7	0.5–40.0	2.6 (18)	2.0	7.4	0.2–10.0	NA			
Drinkable solution, bed rest position	8.4 (16)	5.0	59.3	3.0–30.0	2.0 (16)	2.0	1.7	0.3–5.0	NA			

*NA* not applicable, *n* number of subjects evaluated

**Table 3:** Statistical analysis of the mouth to stomach transit time (MST) as described in Table 1

Administration	MST, s		
	Tablet, bed rest position	Drinkable solution, standing position	Drinkable solution, bed rest position
Tablet, standing position			
x	0.41	0.37	0.43
Md	0.52	0.61	0.31
S	0.09	**0.001**	**0.001**
Tablet, bed rest position			
x		0.11	0.14
Md		0.10	**0.04**
S		**0.001**	**0.001**
Drinkable solution, standing position			
x			0.58
Md			**0.004**
S			0.69

Significance probability was calculated using Student’s *t* test for independent groups for mean (x) and variance (S) and Wilcoxon rank test for median (Md) values

Bold values indicate significantly different

Regarding the MDT, access to the duodenum was quick after swallowing 100 mL of alendronate solution but the difference in access to the duodenum between both positions was not significant (*p* < 0.75). Surprisingly, however, variance was significantly smaller in the group examined in the bed rest position (*p* < 0.05), suggesting that the formulation could be a good option for bedridden patients. Indeed, with the drinkable formulation, the alendronate was available in the intestine in <3 min on average, and no test showed results longer than 10 min. On the other hand, most of the alendronate tablets disintegrated in the stomach as expected (differences between body positions were not significant, *p* < 0.79 for the means and *p* < 0.32 for the variances), including two cases in which the tablet remained intact after dosing for 17 min (in the standing position) and for more than 20 min (in the bed rest position), and passed intact to the duodenum in the latter subject. Three other cases of asymptomatic tablet disintegration in the esophagus were observed. In addition, the only significant influence of age was on the MDT within the group that was administered the drinkable solution in the bed rest position, with *r* = −0.53 (*p* < 0.04; *n* = 11), which indicates that transit might be somewhat slower in older patients. Mean height of the subjects was 167.4 cm (range 152–200 cm) and tended to correlate with the values of MST of the tablet taken in the bed rest position (*r* = 0.51; *p* < 0.07). The body mass index of the subjects did not correlate with any of the transit measurements taken. The MST correlation coefficient was high, *r* = 0.79 (*p* < 0.02; *n* = 8) when comparing the groups who had been administered the tablets and drinkable solution in the bed rest position, suggesting that the transit for both formulations is equally good when subjects have no apparent motility problems. For the MST the group taking the drinkable solution in the bed rest position also correlates moderately with the group taking it in the standing position *r* = 0.67 (*p* < 0.02; *n* = 12). Finally, accessibility to the duodenum with the drinkable solution tends to correlate moderately *r* = 0.55 but not significantly in this study (*p* < 0.07; *n* = 12).

No adverse effects were reported during the study or the week after the study.

## Discussion

We show here that in young men under strictly controlled conditions, the alendronate drinkable solution is bioequivalent to the reference tablets and therefore suitable for the long term treatment of osteoporosis. In young healthy subjects under optimal conditions (precise compliance with dosing instructions, quantity and quality of water consumed, no food intake, proper body position) the pharmacokinetics of the alendronate released from the tablet are equivalent to those of the liquid formulation. As this type of studies are usually done in healthy young volunteers this may limit the conclusions to this group. However, we also showed that there are differences between the two preparations when given to older adults which may have important implications for the treatment of elderly patients in clinical practice. Mean esophageal transit for the drinkable alendronate solution is around 7–8 s, with the mean small bolus transit velocity described in 6 s [24]. With the drinkable solution, the alendronate was delivered in a completely soluble form to the first portion of the intestine in <3 min on average, in both the standing and the lying positions, and the longest time to reach the absorption site was <10 min in all studied subjects, including those with hiatus herniae. Hence, the recommended minimum 30 min postdosing fast is a prudent period to enable further absorption of alendronate at the most suitable site, i.e., the first portion of the intestine. Conversely, although the tablet also disintegrates in <4 min on average, it sometimes remains intact in the stomach and parts of the tablet can even remain in the esophagus. This potential greater variability in the availability of the tablets to the absorption site suggests that alendronate might be absorbed in places were the mucosa is very sensitive. The findings also suggest that the specific instructions for the administration of the alendronate tablets (remaining upright for 30 min after dosing, dosing with NLT 200 mL water) represent only a minimum requirement which is not fully effective in some patients. These considerations are not applicable to the liquid formulation, which is delivered quickly to the intestine irrespective of body position. Because volunteers older than 70 years, who may have more difficulties in swallowing, were not allowed by the CME to participate in the study, we cannot extrapolate the results to this age group.

In addition, the alendronate tablet used in this study was the reference formulation with a fast disintegration time manufactured by Merck Sharp & Dohme Limited, and differences may be greater when generic tablets are used. Although the tablets used here were slightly modified by the test methodology, the fact that some units dissolved early in the esophagus, while others appeared intact in the duodenum seems to indicate that the tablets were neither physically weakened nor strengthened to any considerable extent by the contrast media used in this study. Moreover, the generic alendronate tablets available in the market, even when bioequivalent, have been questioned as a result of potential differences in their in vitro disintegration time, which can be as long as 13 min [25–28]. Such rate suggests that the slower dissolution gradient of the solid generics exposes the digestive walls to protracted high concentrations of alendronate during a critical period, probably affecting the local reactivity of the tissue and the tolerability, or favoring the undesired interactions of the drug with the intradigestive environment and/or contents [29]. So we agree with the authors who claim that even with approved bioequivalence studies, generic tablets may perform differently in practice if their disintegration characteristics and quality are not adequately controlled. A recent article by Kanis et al. [30] report a dissimilar profile of adverse effects, compliance and cost/utility variables when switching to generic tablets of bisphosphonates. In fact, the comparison of formulations under experimental conditions such as bioequivalence studies may mask broader differences that can appear in practice. In the typical bioequivalence test, healthy young adults are recruited and properly trained to follow all dosing instructions, pay attention to the warnings and drink adequate volumes of water. Usually, the postdosing fasting period is strictly controlled by the staff and can be as long as 4 h, during which the influence of delays in transit, disintegration or reflux on the results can be compensated by a certain amount of late absorption [31–36]. In practice such conditions are not at all likely to occur.

The present study shows that in older subjects, administering alendronate via drinkable solution induces fewer variations in the access to the intestine. In agreement with this view, a 6 month placebo-controlled clinical study involving 392 postmenopausal women treated with a different oral solution formulation (alendronate 70 mg/75 mL plus extra water) showed that the urinary NTx corrected for creatinine and serum bone- specific alkaline phosphatase reductions were (95 % confidence interval) −56.1 to −38.8 and −44.9 to −32.6, respectively, a range of variability that is quite narrow for a bisphosphonate and entirely within the effective levels. As expected, the tolerability of the oral solution in such sample was slightly lower than for the placebo, but the rate of upper digestive tract adverse events that were serious or led to discontinuation of treatment was similar [37].

Finally, the pharmaceutical drinkable solution of alendronate taken with no added water as in this study precludes interactions with minerals that can occur when patients use water, whether tap or bottled, to swallow (dissolve) alendronate tablets [38].

The 100 mL volume of the test formulation was enough to enable the alendronate to be available in the intestine rapidly; this agrees with physiological studies of water emptying from the stomach, according to which even smaller volumes can be emptied faster [39]. Moreover, the addition of synthetic viscosity agents to the drinkable solution formulation enables it to progress through the digestive system with some syrup-like properties, that is, to spread a little bit more slowly than water in the stomach and to remain for a longer time in the first portion of the intestine. This thinly viscous mass may make completely soluble alendronate molecules available for a longer time at the proper absorption site. Furthermore, even in cases in which only partial volumes are delivered to the duodenum within the 30 min fasting period, such quantity is enough to allow the expected average absorption of around 1 % of the active principle. Besides volume considerations, the calorie content of the alendronate solution is negligible, and it is probable that if administered after cooling in the refrigerator, transit may be even quicker, improving palatability at the same time [39–41].

Oral solution formulations do not enhance alendronate absorption, as proved by bioequivalence studies vs. the tablets, provided the comparison test has been conducted under adequate experimental conditions, yet the drinkable solution of alendronate is less affected by certain variables in practice, as shown in this study.

The video deglutition radiological test used in this study can be considered in patients treated with alendronate who do not appear to respond or report digestive intolerance. Transit problems with the tablet formulation can be asymptomatic and more frequent than one might expect. Conveniently, as it is the transportation mode and not the molecule that is the cause of the unsatisfactory result, patients should remain under treatment with alendronate and change the vehicle of administration before switching to a different active compound which might be less effective, have uncertain safety parameters or be more expensive.

In conclusion, the alendronate solution has rapid access to the absorption site and is less subject to transit problems than the tablet formulation. Therefore, alendronate, the drug of choice for the treatment of osteoporosis in many countries, can be administered on weekly basis in an optimized manner even in bedridden patients.
